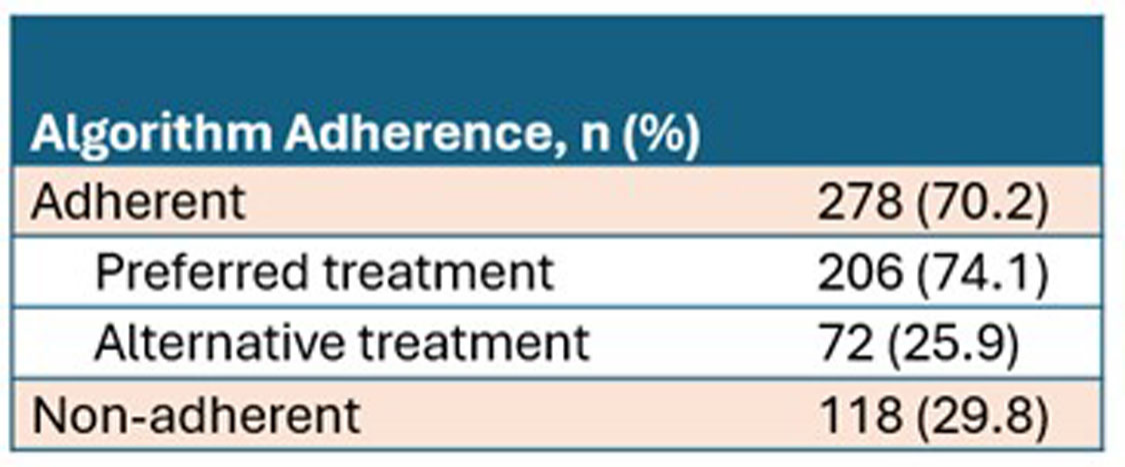# 142 Diagnostic Stewardship of Urine Testing Through Clinical Decision Support: An Interrupted Time Series Analysis

**DOI:** 10.1017/ash.2026.10548

**Published:** 2026-06-23

**Authors:** Marianna Almpani, Daniel O’Connor, Jennifer Do, Jessica Lomanno, Theodore (Ted) Rader

**Affiliations:** 1 UMass; 2 UMass Memorial Medical Center

## Abstract

**Background:** Clostridioides difficile infection (CDI) remains one of the most common healthcare-associated infections and contributes significantly to morbidity, mortality, and healthcare costs. In 2021, the Infectious Diseases Society of America (IDSA) and the Society for Healthcare Epidemiology of America (SHEA) updated their clinical practice guidelines for CDI treatment, recommending fidaxomicin over vancomycin for both initial and recurrent episodes due to comparable cure rates and a lower risk of recurrence. This recommendation is contingent upon institutional resource availability, as fidaxomicin is currently substantially more expensive than vancomycin. In response to these updated guidelines, a treatment algorithm was developed at UMass Memorial Medical Center (UMMMC) to prioritize fidaxomicin use in patients at high risk for recurrence (Figure 1). This study evaluated the adherence to and clinical outcomes of the institutional CDI treatment algorithm following implementation. Methods This retrospective study evaluated 530 patients admitted to UMMMC with a positive C. difficile PCR test during that admission between January 1, 2024, and June 30, 2025. Of those, 396 met inclusion criteria (Figure 2). The primary outcome was clinical cure, defined as resolution of diarrhea without the need for additional treatment within two days following completion of therapy. Secondary outcomes included 30-day mortality, recurrence at 30 and 90 days, hospital length-of-stay, and treatment-related adverse effects. Statistical analysis was performed using chi-square test for categorical variables, or one-way analysis of variance (ANOVA) for continuous variables, with Bonferroni post-hoc testing in GraphPad Prism. Results No significant differences were observed between treatment groups for the primary outcome of clinical cure (Table 1). Similarly, there were no significant differences in secondary outcomes, including recurrence at 30 and 90 days, or hospital length of stay (Table 1). There was no 30-day mortality documented for any of the groups. No adverse effects associated with vancomycin or fidaxomicin therapy were documented. The majority of treatment decisions were adherent to the institutional algorithm (70.2%) (Table 2). Conclusion Restricting fidaxomicin use to CDI patients at high risk for recurrence appears to be a safe and effective approach. This treatment algorithm provides a practical framework that may be adopted by other institutions to balance cost and efficacy, particularly as the high cost of fidaxomicin remains a barrier for both patients and healthcare systems.

**Figure:**
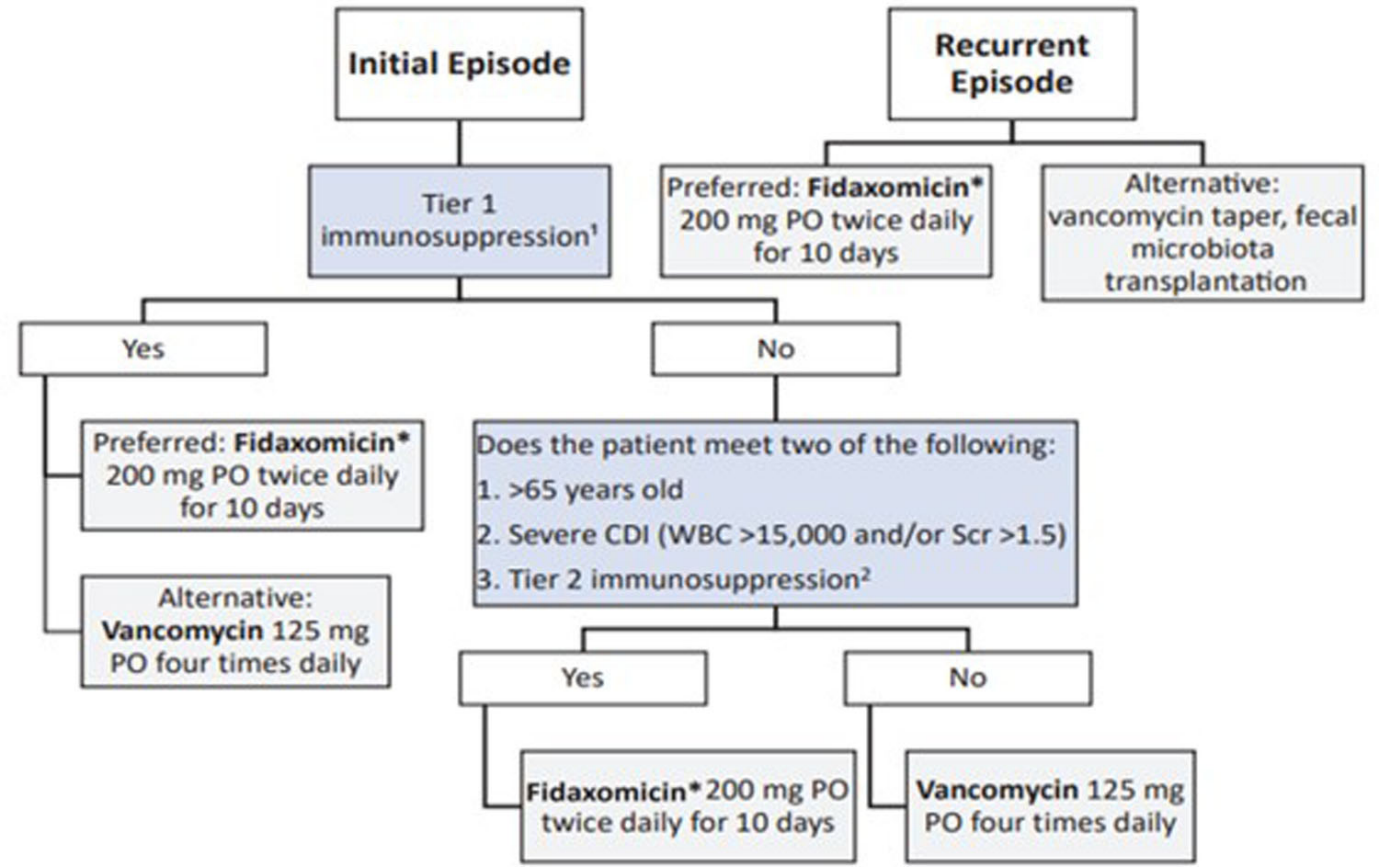

**Figure:**
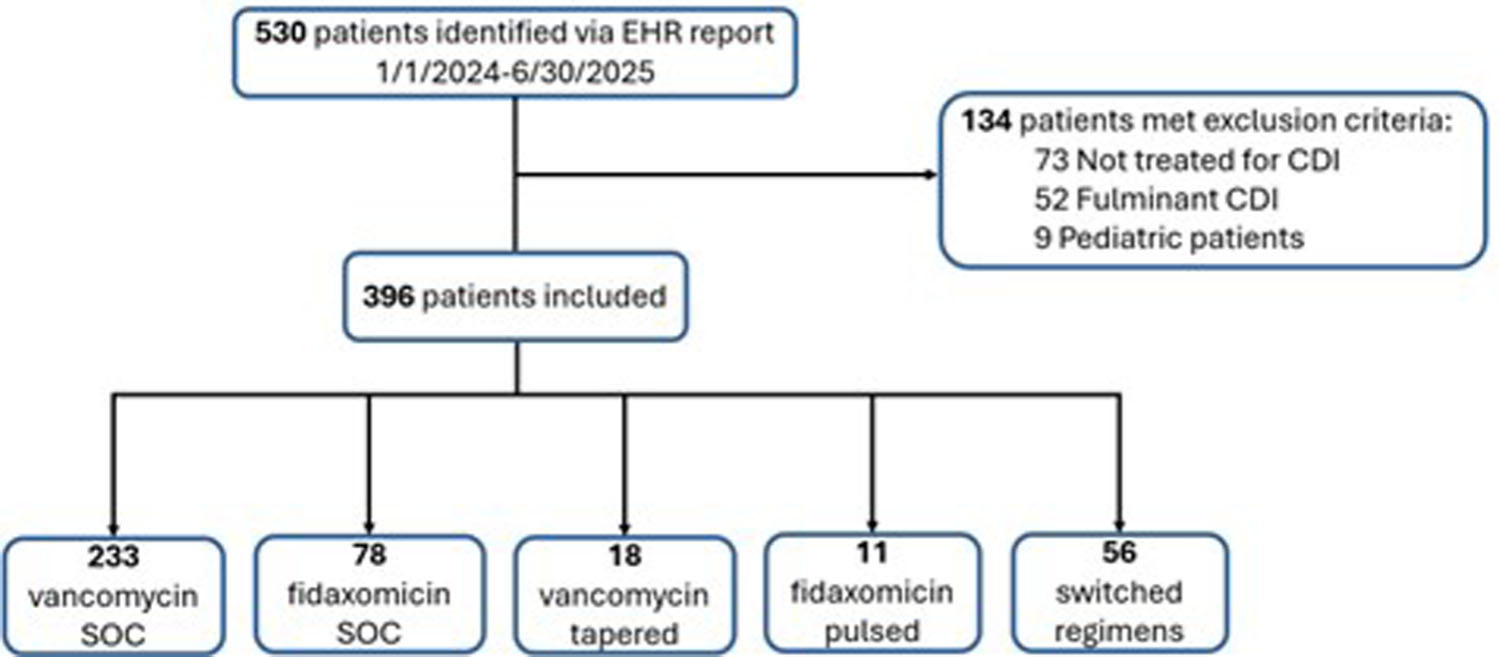

**Figure:**
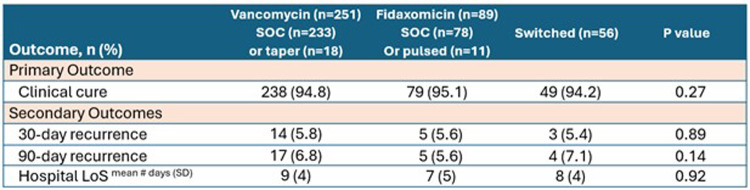

**Figure:**